# Acceptance of COVID-19 Vaccination during the COVID-19 Pandemic in China

**DOI:** 10.3390/vaccines8030482

**Published:** 2020-08-27

**Authors:** Jiahao Wang, Rize Jing, Xiaozhen Lai, Haijun Zhang, Yun Lyu, Maria Deloria Knoll, Hai Fang

**Affiliations:** 1 School of Public Health, Peking University, Beijing 100083, China; jiahaowang@pku.edu.cn (J.W.); rzjing2015@hsc.pku.edu.cn (R.J.); laixiaozhen@pku.edu.cn (X.L.); haijunzhang@pku.edu.cn (H.Z.); lydialu1217@hotmail.com (Y.L.); 2 China Center for Health Development Studies, Peking University, Beijing 100083, China; 3 International Vaccine Access Center, Bloomberg School of Public Health, Johns Hopkins University, Baltimore, MD 21205, USA; mknoll2@jhu.edu; 4 Peking University Health Science Center-Chinese Center for Disease Control and Prevention Joint Center for Vaccine Economics, Beijing 100083, China; 5 Key Laboratory of Reproductive Health, National Health Commission of the People’s Republic of China, Beijing 100083, China

**Keywords:** SARS-CoV-2, COVID-19, immunization, vaccine acceptance, vaccine preference

## Abstract

Background: Faced with the coronavirus disease 2019 (COVID-19) pandemic, the development of COVID-19 vaccines has been progressing at an unprecedented rate. This study aimed to evaluate the acceptance of COVID-19 vaccination in China and give suggestions for vaccination strategies and immunization programs accordingly. Methods: In March 2020, an anonymous cross-sectional survey was conducted online among Chinese adults. The questionnaire collected socio-demographic characteristics, risk perception, the impact of COVID-19, attitudes, acceptance and attribute preferences of vaccines against COVID-19 during the pandemic. Multivariate logistic regression was performed to identify the influencing factors of vaccination acceptance. Results: Of the 2058 participants surveyed, 1879 (91.3%) stated that they would accept COVID-19 vaccination after the vaccine becomes available, among whom 980 (52.2%) wanted to get vaccinated as soon as possible, while others (47.8%) would delay the vaccination until the vaccine’s safety was confirmed. Participants preferred a routine immunization schedule (49.4%) to emergency vaccination (9.0%) or either of them (41.6%). Logistic regression showed that being male, being married, perceiving a high risk of infection, being vaccinated against influenza in the past season, believing in the efficacy of COVID-19 vaccination or valuing doctor’s recommendations could increase the probability of accepting COVID-19 vaccination as soon as possible, while having confirmed or suspected cases in local areas, valuing vaccination convenience or vaccine price in decision-making could hinder participants from immediate vaccination. Conclusion: During the pandemic period, a strong demand for and high acceptance of COVID-19 vaccination has been shown among the Chinese population, while concerns about vaccine safety may hinder the promotion of vaccine uptake. To expand vaccination coverage, immunization programs should be designed to remove barriers in terms of vaccine price and vaccination convenience, and health education and communication from authoritative sources are important ways to alleviate public concerns about vaccine safety.

## 1. Introduction

The coronavirus disease 2019 (COVID-19) pandemic has imposed a heavy disease burden around the world, and there are currently no specific antiviral treatments for COVID-19 [1,2,3]. As immunization is one of the most successful and cost-effective health interventions to prevent infectious diseases, vaccines against COVID-19 are considered to be of great importance to prevent and control COVID-19 [4,5]. Countries worldwide are trying to accelerate the research and development of COVID-19 vaccines, and it has been reported that there have been more than 160 candidate vaccines to date, with around 20 candidates in clinical evaluation [4,6].

Although great progress has been made, there are still important challenges regarding future immunization against COVID-19, one of which is the uncertainty about the public acceptance of COVID-19 vaccination. Vaccine acceptance reflects the overall perception of disease risk, vaccine attitudes and demand within the general population, which is critical for the success of immunization programs to attain high vaccination coverage rates, especially for newly emerging infectious diseases [7,8,9]. Reports on the acceptance and uptake of pandemic vaccines, such as for the 2009 H1N1 pandemic, have shown unsatisfying results, as the willingness to receive the 2009 H1N1 pandemic vaccine among the general public ranged from 17% to 67% across studies from Australia, America, Greece, the UK and France [10,11,12,13,14,15,16,17]. Previous studies on vaccine acceptance and theories of health behavior, such as the health belief model or protection motivation theory, have identified many factors that influence the acceptance or uptake of a pandemic vaccine, including the risk perception of the disease, perception of vaccine safety and efficacy, general vaccination attitude, past vaccination history, recommendations from doctors, price, vaccination convenience and socio-demographic characteristics [7,9,11,12,14,15,17,18,19,20,21,22].

In addition to the problem of unsatisfactory acceptance, the real uptake rate of pandemic vaccines could be much lower than the acceptance after the introduction of the vaccine and promotion of mass immunization programs [12,15,23,24]. For example, only 10% of the population received a vaccination at the end of the domestic H1N1 outbreak in France compared to the vaccination intention of 17% or 27.4% [12,15,24]. Even in high-risk populations such as healthcare workers, only 25% received the pandemic H1N1 vaccination when it was provided for free in Beijing, China [23]. The hesitancy or delay regarding vaccination was the primary reason for this discrepancy, even among those who had intended to get a vaccination [9,10,25,26]. Recent articles have found that some impact factors on vaccination acceptance helped to explain vaccination hesitancy or vaccination delay behavior, and the cultural, social or political differences across countries should also be considered in the vaccination decision-making process [9,18,27,28]. For novel vaccines against new emerging pandemics, such as the 2009 H1N1 pandemic, public concern about vaccine safety was frequently identified as a serious barrier to vaccine acceptance [7,10,13,14,17,25], while attitudes and past history regarding vaccination—especially influenza vaccination history—were the major predictors of pandemic vaccine uptake [7,10,11,12,15,17,18]. As the COVID-19 pandemic has shown higher severity in terms of transmissibility and mortality compared with past pandemics of influenza, countries around the world—including China—are facing great pressure to control the current pandemic and prevent a possible recurrence of damaging waves or epidemics in the future. In this case, understanding the influencing factors of the acceptance of COVID-19 vaccination and identifying common barriers and facilitators for vaccination decisions are important aspects in the design of effective strategies to improve the vaccine coverage rate among the general population [9,29,30,31,32,33].

This study aimed to evaluate the acceptance of future COVID-19 vaccination, the preference for vaccine attributes and vaccination schedules, as well as the influencing factors on vaccination acceptance among the Chinese adult population. This information is critical to preparing well for future vaccination strategies and immunization programs against COVID-19.

## 2. Materials and Methods 

### 2.1. Study Design, Population and Sampling

In March 2020, an anonymous cross-sectional survey was conducted online using a stratified random sampling method on the largest online survey platform in China: Wen Juan Xing (Changsha Ranxing Information Technology Co., Ltd., Hunan, China). Wen Juan Xing, equivalent to Qualtrics, SurveyMonkey or CloudResearch, provides online questionnaire design and survey functions for enterprises, research institutions and individuals. The Wen Juan Xing sample database covers over 2.6 million respondents, whose personal information was confirmed, allowing for an authentic, diverse and representative sample. The target population in the present study was Chinese adults living in Mainland China; thus, a random sample procedure stratified by age and location was adopted to match Chinese adults in the Wen Juan Xing sample database. Chinese respondents aged 18 years and above residing in Mainland China on the Wen Juan Xing sample database were eligible to participate in the survey. In general, 2100 respondents were randomly selected, and the final sample consisted of 2058 respondents after quality control and manual check procedures to exclude incomplete and invalid questionnaires. The study was approved by Peking University Institutional Review Board (IRB00001052-20011). 

### 2.2. Measures

The self-administered questionnaire was designed based on previous studies and frameworks to assess vaccine acceptance for newly emerging infectious diseases such H1N1 or Ebola [10,11,13,14,15,17,18,19,34,35]. The contents of the questionnaire included (1) socio-demographic characteristics, such as age, sex, marital status, education, employment status, family income and health status; (2) perceived perception of risk for the COVID-19 pandemic; (3) the impact of the COVID-19 pandemic on respondents’ work/study, income and daily life; (4) vaccination history, such as seasonal influenza vaccination in the past season; (5) acceptance, attitude, vaccination preferences for future COVID-19 vaccination and the importance of identified impact factors on the respondents’ vaccination decision-making, such as vaccine price, convenience and doctor’s recommendations. All questions were closed-ended, with tick boxes provided for responses. Most questions were treated as categorical variables, and self-reported questions were assessed on a five-point Likert scale, such as health status, perceived risk of infection and impact of the COVID-19 pandemic on respondents. 

### 2.3. Statistical Analysis

The primary outcome of the survey was the acceptance of COVID-19 vaccination. Respondents who chose “yes” to the question “If a COVID-19 vaccine is successfully developed and approved for listing in the future, would you accept vaccination?” were classified into the accept group, while those who chose “no” were assigned to the refuse group. We further categorized those in the accept group into the vaccine demand group or vaccine delay group using the question “Do you want to be vaccinated as soon as possible when the COVID-19 vaccine is available?” Those who wanted to get vaccinated as soon as possible were included in the vaccine demand group, and others who wanted to delay the vaccination until the vaccine safety was confirmed were included in the vaccine delay group.

Descriptive statistics were performed to describe the socio-demographic characteristics, risk perception, pandemic impact, acceptance, attitudes and preferences of vaccine attributes and vaccination schedules of the future COVID-19 vaccine. Response categories of the pandemic impact were sorted into three groups, and the responses of health status were categorized into two groups. Those who skipped questions regarding the pandemic’s impact due to their employment status were defined as missing in each question. The information of family income was surveyed in Chinese yuan (CNY) and also presented in US dollars (USD) at an exchange rate of 6.9 yuan per dollar in 2020. The baseline characteristics were compared between respondents in the two groups (vaccine demand group vs. vaccine delayed group), with the chi-squared test to analyze the significance of the association between categorical variables. Multivariate logistic regression was then performed between the vaccine demand group and vaccine delay group to identify the influencing factors of vaccination acceptance (immediate or delayed acceptance), with the odds ratio (OR), standard error (SE) and a 95% confidence interval (CI) being calculated. All data were analyzed using STATA, version 14.0 (Stata Corp, College Station, TX, USA). 

## 3. Results

### 3.1. Study Sample Characteristics 

**Figure 1:**
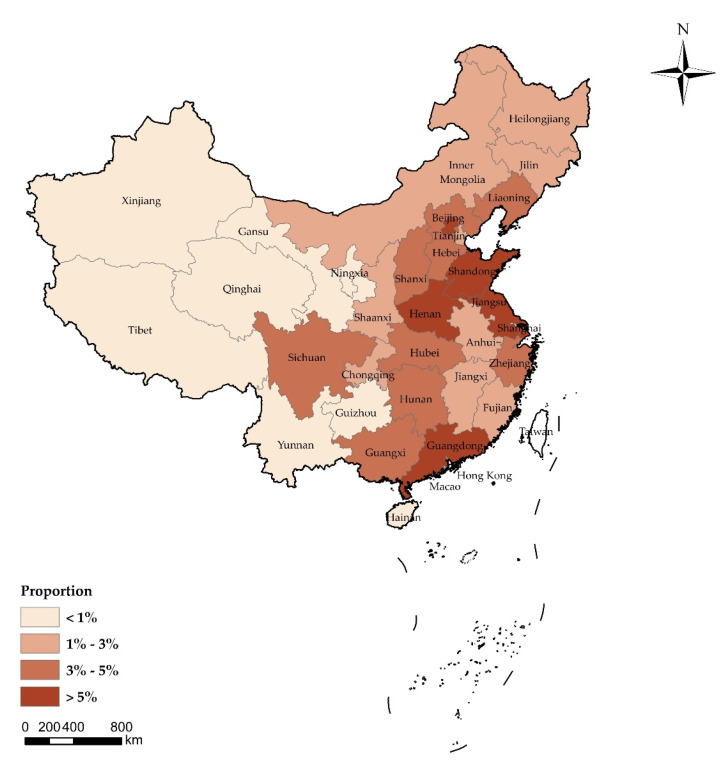
The provincial distribution of respondents (n = 2058) of the survey on the acceptance of COVID-19 vaccination in China.

In total, 2058 out of 2100 respondents completed the questionnaires, with a response rate of 98.0%. Figure 1 shows the provincial distribution of respondents, and Table 1 presents the basic characteristics, risk perception, impact of COVID-19 and vaccination history of respondents. In general, respondents were located in all 31 provincial administrative regions of Mainland China (Appendix A Table A1). Half of them (50.2%) were between 31 and 50 years old, and 7.3% (n = 150) were more than 51 years old. In addition, 54.2% were female, 67.3% were married and 80.2% were employed. Regarding education background, 38.2% had a high school and below level of education and 55.4% had an associate or bachelor’s degree. In terms of location, 58.1% were located in Eastern China and 79.6% lived in urban areas. In total, 74.2% thought that their health status was good or very good. The majority of respondents (51.2%) had a total family income in 2019 ranging from CNY 50,000 to CNY 150,000 (USD 7246 to 21,739).

During the survey, 74.7% of respondents stated that there were confirmed or suspected cases in the county in which they lived, but only 12.2% perceived the risk of COVID-19 infection as high or very high. The pandemic has affected respondents on a large scale, as 66.5%, 64.4% and 44.0% of respondents thought that the impact of pandemic on their daily life, work and income was large or very large, respectively. In terms of vaccination history, 14.6% of respondents have received vaccinations against influenza in the past season, while 22.3% reported that they have ever refused vaccination with one or more types of vaccines previously.

### 3.2. Acceptance, Preferences and Impact Factors of the Future COVID-19 Vaccine

Table 2 presents the acceptance of the future COVID-19 vaccine and its impact factors among all respondents, as well as vaccination preferences in the vaccine accept group. Of the total 2058 respondents, 1842 (89.5%) thought that vaccination would be an effective way to prevent and control COVID-19, and 1879 (91.3%) would accept vaccination if the COVID-19 vaccine were successfully developed and approved for listing in the future. In terms of the importance of some factors in vaccination decision-making, the majority considered that their doctor’s recommendation (80.6%) or vaccine convenience (75.7%) was an important factor affecting their vaccination intention. Over half of the respondents (59.9%) thought that the vaccine price was important. Among the 1879 respondents in the vaccine accept group, 52.2% wanted to get vaccinated as soon as possible when it becomes available, while others (47.8%) would delay vaccination until they could confirm the vaccine’s safety. Most respondents preferred to receive vaccinations with routine immunization schedules in advance of the epidemic (49.4%) rather than an emergency vaccination (9.0%) or accepting both immunization schedules (41.6%). In total, 64.2% showed no preference for domestic or imported vaccines, while 32.5% would prefer a domestic vaccine.

### 3.3. Influencing Factors of Vaccination Acceptance

As the majority of respondents (91.3%) stated that they would accept COVID-19 vaccination, multivariate logistic regression was then preformed between the vaccine demand group (n = 980) and vaccine delay group (n = 899) to identify the influencing factors of vaccination acceptance (immediate or delayed acceptance). The comparison of baseline characteristics between the two groups with chi-squared tests was displayed in Table S1. Socio-demographic characteristics, risk perception, impact of COVID-19, vaccination history, attitude towards COVID-19 and impact factors in decision-making were included in the regression, with the vaccine delay group as the reference group (See Table 3). Besides, Table S2 presents the results of regression, including significant factors at the 10% level of chi-squared tests.

Among those who would accept vaccination, male (odds ratio (OR):1.25, 95% confidence interval (CI): 1.03–1.52) or married (OR:1.70, 95% CI: 1.26–2.29) respondents were more likely to accept COVID-19 vaccination as soon as possible. Moreover, those perceiving a high or very high risk of infection (OR:1.46, 95% CI: 1.04–2.05), who had been vaccinated against influenza in the past season (OR:1.90, 95% CI: 1.43–2.51), who believed that COVID-19 vaccination is an effective way to prevent and control COVID-19 (OR:1.56, 95% CI: 1.08–2.25) or who valued doctor’s recommendation as an important factor in vaccination decision-making (OR:2.32, 95% CI: 1.76–3.07) also tended to accept COVID-19 vaccination as soon as possible. In contrast, those with confirmed or suspected cases in the county in which they lived (OR:0.72, 95% CI: 0.57–0.91) and who considered that vaccination convenience (OR:0.69, 95% CI: 0.54–0.89) or vaccine price (OR:0.75, 95% CI: 0.61–0.93) were important factors in vaccination decision-making were less likely to accept vaccination as soon as possible.

## 4. Discussion

The present study reported high acceptance of COVID-19 vaccination among the Chinese population during the COVID-19 pandemic. Most (91.3%) of the participants stated that they intended to receive COVID-19 vaccination if it was developed successfully and approved for listing in the future. More than half (52.2%) of respondents in the vaccine accept group wanted to get vaccinated as soon as possible when it was available, while others (47.8%) would delay the vaccination until they confirmed the vaccine’s safety. In terms of vaccination preferences, the majority thought that both immunization schedules (routine or emergency immunization) and both types of vaccine (domestic or imported) were acceptable, while a routine immunization schedule and the domestic vaccine were more frequently preferred. Among respondents who accepted vaccination, significant factors influencing their vaccination acceptance were gender, marriage status, risk perception, influenza vaccination history, belief of COVID-19 vaccine efficacy, valuing doctor’s recommendations, vaccination convenience or vaccine price.

The high acceptance of and positive attitude toward COVID-19 vaccination among the Chinese population reflected the strong demand for the vaccine and the high recognition of the importance of vaccines in controlling pandemics. The acceptance of COVID-19 vaccination in China was higher than that of the H1N1 vaccine, not only in other countries and regions, which ranged from 17% to 67% [7,10,11,12,13,14,15,16,17,18,19], but also in China, which was estimated to have a roughly 37.3% H1N1 pandemic influenza vaccination rate [23]. This reflects the difference in public perception about the two pandemics in terms of the disease severity, infection risk, vaccine importance or attitude, as well as some macro-level factors such as social or cultural factors across countries [9,18]. As reported in this study, the pandemic has had a profound impact on the work, income or daily life of Chinese residents. To address these challenges, China has taken drastic measures and public health interventions to control the transmission of COVID-19 since the outbreak of the disease, and these actions have substantially mitigated the spread of COVID-19 [32,33,36]. Therefore, although 74.7% of respondents reported having confirmed or suspected cases in the county in which they lived, only a small portion (12.2%) perceived a high or very high risk of the disease. In comparison, we found that Chinese residents held strong beliefs about the efficacy of COVID-19 vaccination, as 89.5% thought that vaccination is an effective way to prevent and control COVID-19, even though the vaccine is still under development. This positive attitude towards COVID-19 vaccination and the large perceived pandemic impact may explain the high acceptance of COVID-19 vaccination among Chinese adults, as they perceive strong benefits from vaccination compared with the risk according to the health belief model [15,18,20,21,37,38]. Moreover, our survey collected respondents’ preferences for immunization schedules and vaccine production and found that they either preferred a routine immunization schedule (49.4%) or accepted both schedules (41.6%) and accepted both domestic and imported vaccines (64.2%). All these findings represented a good start in the process of achieving the coverage rate required to ensure herd immunity among the Chinese population, and the acceleration of COVID-19 vaccine development and listing for application for the public is therefore urged in response to the COVID-19 pandemic at this stage [4,39].

Although a high acceptance rate has been observed, there are still some barriers in the process of moving from the vaccination intention to real uptake behavior. Around half of respondents (47.8%) with vaccination intention would delay vaccination until the safety of the vaccine is confirmed, and concerns or uncertainty about vaccine safety led to their vaccine hesitation. Public concern about vaccine safety has frequently been reported as the major obstacle to vaccination decision-making, especially for newly introduced vaccines which have not been fully tested in the real world [7,8,9,12,13,14,17,18,19,25,27,28]. For example, 13% of Australian people stated that they would wait to see if there were any adverse events before agreeing to get vaccinated, while their acceptance rate was as high as 67% [10]. Two reasons may explain the vaccine delay observed in this study: firstly, the vaccine against COVID-19 was still under development during the survey period, and there was no information about vaccine safety for reference; secondly, concerns about using new vaccines during a pandemic were reported to differ from those of established products in a non-crisis situation, as the uncertainties about new vaccines, new emerging infectious diseases and concerns about the pharmaceuticals would lower the vaccine confidence of the public [25]. However, the hesitancy that our study reported may be reduced later, when a vaccine becomes available, as a quantitative study in Dutch reported that, once the safety of newly-introduced vaccines becomes comparable to that of vaccines which are already on the market, the importance of this factor on vaccination decision-making becomes less than other attributes such as vaccine effectiveness or cost [22]. This also requires an increased focus on vaccine safety in the research and development of new vaccines and better performance in terms of many other vaccine attributes such as price and convenience to increase vaccine uptake, especially for those who accept vaccination but with hesitation.

As the majority (91.3%) of respondents had the intention of getting vaccinated, it is meaningful to identify other barriers or facilitators to their vaccination decision on whether to accept vaccination as soon as possible. The risk perception of respondents was an important predictor for vaccination acceptance, as those who perceived a high or very high risk of infection were more likely to get vaccinated as soon as possible instead of delaying it. Our study also confirmed the positive role of influenza vaccination history and belief in vaccine effectiveness in accepting immediate vaccination, which was consistent with previous studies [7,10,11,12,15,17,18]. Furthermore, we found that those who valued doctor’s recommendations tended to get vaccinated immediately, while those who valued vaccination convenience or vaccine price in decision-making tended to choose delayed vaccination. Inconsistent results were shown in previous studies from the UK, France or Australia concerning the impact of socio-demographic characteristics on the acceptance of a pandemic vaccine [7,12,16,17], and we found that, among the Chinese population, male or married respondents were more likely to accept immediate vaccination against the pandemic, while respondents’ education or income may not influence their intention. Our findings are useful for designing effective vaccination strategies and immunization programs for those with vaccine hesitancy. Firstly, the vaccine price should be affordable for the public, and it is promising that China has stated that it aims to make its COVID-19 vaccine a global public good when it is ready for application [22,26,40]. Secondly, measures should be taken to increase vaccine convenience and accessibility in terms of vaccine manufacture, distribution, supply, immunization service, etc. [9,22,27]. Last but not least, monitoring information about vaccine safety should be made public on a regular basis after the application of the vaccine, and timely health education and communication conducted by authoritative sources such as healthcare professionals will be critical to alleviate public concerns about vaccine safety [9,27]. In response to future possible pandemics, health departments and other sectors should consider regular vaccination and education programs for established vaccines for non-pandemic infectious diseases such as influenza to improve overall vaccine confidence and the compliance of the public [9,26,28].

This is the first study to investigate the acceptance of COVID-19 vaccination among a large population in China during the COVID-19 pandemic period, which provided baseline information for the ongoing monitoring of the acceptance of COVID-19 vaccination by public. By considering the vaccine hesitancy and dividing respondents based on acceptance levels (refused, immediate or delayed acceptance), our study contributed to the comparison within countries about the influencing factors of pandemic vaccination acceptance, such as some socio-demographic characteristics. The exploration of barriers and facilitators of vaccination was useful for identifying priority groups that need special attention in the vaccination campaign and helped to design effective immunization strategies to increase the vaccine uptake in the prevention and control of the COVID-19 pandemic. Our study has several limitations. First of all, as the use of an offline survey was not feasible during the pandemic period, the online survey may limit the representativeness of the present study’s sample. To address this problem, we enrolled a large sample size and used a random sampling method stratified by demographic characteristics to increase the sample diversity and representativeness. Secondly, given the hypothetical nature, the study results may differ from real practice, and some self-reported answers may lead to information bias. Further studies are needed to investigate the acceptance of COVID-19 vaccination in different periods of the pandemic and to take vaccine efficiency and safety into consideration after the vaccine is made available to the public.

## 5. Conclusions

This study reflected a high level of acceptance of COVID-19 vaccination among the adult population in China during the pandemic period. Concerns about vaccine safety by the public may hinder the promotion of vaccine uptake in the future. To expand vaccine uptake in response to the COVID-19 pandemic, immunization programs should be designed to remove barriers in vaccine price and vaccination convenience. In addition, health education and communication from authoritative sources will be important to alleviate public concerns about vaccine safety.

## Figures and Tables

**Table 1:** The basic characteristics, risk perception, impact of COVID-19 and vaccination history of the 2058 respondents in the survey.

Items	Respondents (n = 2058) N (%)
Age group	
18–25	475 (23.1)
26–30	400 (19.4)
31–40	523 (25.4)
41–50	510 (24.8)
51 and above	150 (7.3)
Gender	
Female	1115 (54.2)
Male	943 (45.8)
Highest level of education	
Middle school and below	123 (6.0)
High school	663 (32.2)
Associate or bachelor	1140 (55.4)
Master and above	132 (6.4)
Marriage status	
Married	1385 (67.3)
Others (single, divorced or widowed)	673 (32.7)
Location	
Central	531 (25.8)
East	1195 (58.1)
West	332 (16.1)
Region	
Rural	420 (20.4)
Urban	1638 (79.6)
Employment status	
Employed	1651 (80.2)
Unemployed	407 (19.8)
Health status	
Good and above (good, very good)	1527 (74.2)
Fair or below (fair, poor, very poor)	531 (25.8)
Total family income in 2019	
≤CNY 50,000 (USD 7246)	277 (13.4)
CNY 50,000–100,000 (USD 7246–14,492)	548 (26.6)
CNY 100,000–150,000 (USD 14,492–21,739)	506 (24.6)
CNY 150,000–200,000 (USD 21,739–28,986)	352 (17.1)
CNY 200,000–300,000 (USD 28,986–43,478)	239 (11.7)
CNY 300,000 (USD 43,478)	136 (6.6)
There are confirmed or suspected cases in the county	
Yes	1538 (74.7)
No or not clear	520 (25.3)
Perceived risk of infection	
High or very high	251 (12.2)
Fair	575 (27.9)
Low or very low	1232 (59.9)
Pandemic impact on daily life	
Large or very large	1368 (66.5)
Fair	497 (24.1)
Small or very small	193 (9.4)
Pandemic impact on work	
Large or very large	1326 (64.4)
Fair	402 (19.5)
Small or very small	191 (9.3)
Missing	139 (6.8)
Pandemic impact on income	
Large or very large	905 (44.0)
Fair	467 (22.7)
Small or very small	325 (15.8)
Missing	361 (17.5)
Received vaccination against influenza in the past season	
Yes	301 (14.6)
No	1757 (85.4)
Refused vaccination of a certain type of vaccine in the past	
Yes	459 (22.3)
No	1599 (77.7)

**Table 2:** Acceptance, preferences and impact factors of the future COVID-19 vaccine for the 2058 respondents in the survey.

Items	N (%)
**Overall respondents (n = 2058)**	
COVID-19 vaccination is an effective way to prevent and control COVID-19	
Yes	1842 (89.5)
No	216 (10.5)
Accept vaccination if the COVID-19 vaccine is successfully developed and approved for listing in the future	
Yes	1879 (91.3)
No	179 (8.7)
Doctor’s recommendation is an important factor in vaccination decision-making	
Yes	1659 (80.6)
No	399 (19.4)
Vaccine convenience (vaccination method, frequency, distance to vaccination sites, etc.) is an important factor in vaccination decision-making	
Yes	1558 (75.7)
No	500 (23.3)
Vaccine price is an important factor in vaccination decision-making	
Yes	1233 (59.9)
No	825 (40.1)
**Vaccine accept group (N = 1879)**	
Want to receive vaccination as soon as possible when the vaccine is available	
Yes, as soon as possible	980 (52.2)
No, delay vaccination until I confirmed the vaccine safety	899 (47.8)
Prefer which kind of immunization schedules of the COVID-19 vaccination	
Routine immunization	928 (49.4)
Emergency vaccination	169 (9.0)
Both are acceptable	782 (41.6)
Prefer which type of COVID-19 vaccines	
Domestic vaccine	611 (32.5)
Imported vaccine	62 (3.3)
Both are acceptable	1206 (64.2)

**Table 3:** Influencing factors on vaccination acceptance between the vaccine demand group and vaccine delay group.

Characteristics	OR	SE	*p*-Value	95% CI
Age group				
18–25	Ref			
26–30	1.15	0.23	0.48	0.78–1.70
31–40	1.00	0.21	0.99	0.66–1.52
41–50	1.05	0.22	0.81	0.69–1.60
>51	1.65	0.48	0.09	0.93–2.92
Gender				
Female	Ref			
Male	1.25	0.13	0.03	1.03–1.52
Highest level of education				
Middle school and below	Ref			
High school	1.11	0.26	0.67	0.70–1.75
Associate or Bachelor	1.27	0.31	0.33	0.79–2.04
Master and above	1.09	0.34	0.79	0.59–2.00
Marriage status				
Others (single, divorced or widowed)	Ref			
Married	1.70	0.26	<0.001	1.26–2.29
Location				
Central	Ref			
East	0.88	0.11	0.29	0.69–1.11
West	1.03	0.16	0.84	0.76–1.40
Region				
Rural	Ref			
Urban	0.84	0.11	0.18	0.65–1.09
Employment status				
Unemployed	Ref			
Employed	1.03	0.51	0.96	0.39–2.7
Health status				
Fair or below (fair, poor, very poor)	Ref			
Good and above (good, very good)	1.16	0.14	0.21	0.92–1.46
Total family income in 2019				
≤CNY 50,000 (USD 7246)	Ref			
CNY 50,000–100,000 (USD 7246–14,492)	0.84	0.15	0.32	0.60–1.18
CNY 100,000–150,000 (USD 14,492–21,739)	0.73	0.13	0.08	0.51–1.04
CNY 150,000–200,000 (USD 21,739–28,986)	0.82	0.16	0.32	0.56–1.21
CNY 200,000–300,000 (USD 28,986–43,478)	0.82	0.18	0.38	0.53–1.27
≥CNY 300,000 (USD 43,478)	1.05	0.27	0.85	0.64–1.73
There are confirmed or suspected cases in the county				
No or not clear	Ref			
Yes	0.72	0.09	0.01	0.57–0.91
Perceived risk of infection				
Fair	Ref			
High or very high	1.46	0.25	0.03	1.04–2.05
Small or very small	1.02	0.12	0.89	0.81–1.27
Pandemic impact on daily life				
Fair	Ref			
Large or very large	1.02	0.13	0.90	0.80–1.30
Small or very small	0.69	0.14	0.07	0.46–1.04
Pandemic impact on work				
Fair	Ref			
Large or very large	1.03	0.15	0.82	0.78–1.36
Small or very small	0.88	0.19	0.55	0.57–1.34
Pandemic impact on income				
Fair	Ref			
Large or very large	0.96	0.13	0.74	0.74–1.24
Small or very small	0.81	0.14	0.20	0.58–1.12
Received vaccination against influenza in the past season				
No	Ref			
Yes	1.90	0.27	<0.001	1.43–2.51
Refused vaccination of a certain type of vaccine in the past				
No	Ref			
Yes	0.81	0.10	0.09	0.64–1.03
COVID-19 vaccination is an effective way to prevent and control COVID-19				
No	Ref			
Yes	1.56	0.29	0.02	1.08–2.25
Doctor’s recommendation is an important factor in vaccination decision-making				
No	Ref			
Yes	2.32	0.33	<0.001	1.76–3.07
Vaccine convenience (vaccination method, frequency, distance to vaccination sites, etc.) is an important factor in vaccination decision-making				
No	Ref			
Yes	0.69	0.09	<0.001	0.54–0.89
Vaccine price is an important factor in vaccination decision-making				
No	Ref			
Yes	0.75	0.08	0.01	0.61–0.93

Notes: Socio-demographic characteristics, risk perception, impact of COVID-19, vaccination history, attitude towards COVID-19 and impact factors in decision-making were included in the regression, with the vaccine delay group as the reference group. OR: odds ratio. SE: standard error. CI: confidence interval.

## Supplementary Materials

The following are available online at https://www.mdpi.com/2076-393X/8/3/482/s1, Table S1: Comparison of baseline characteristics between the vaccine demand group and vaccine delay group, Table S2. Influencing factors on vaccination acceptance between the vaccine demand group and vaccine delay group, including significant factors at the 10% level of the chi-squared test in the multivariate logistic regression.

## Appendix A

**Table A1:** The provincial distribution of respondents (n = 2058) in the survey on the acceptance of COVID-19 vaccination in China.

Provincial Administrative Regions	Respondents (N = 2058)
	N (%)
Beijing	138 (6.7)
Tianjin	37 (1.8)
Hebei	96 (4.7)
Shanxi	68 (3.3)
Inner Mongolia	24 (1.2)
Liaoning	69 (3.4)
Jilin	24 (1.2)
Heilongjiang	38 (1.8)
Shanghai	146 (7.1)
Jiangsu	130 (6.3)
Zhejiang	88 (4.3)
Anhui	62 (3.0)
Fujian	62 (3.0)
Jiangxi	38 (1.8)
Shandong	129 (6.3)
Henan	134 (6.5)
Hubei	99 (4.8)
Hunan	68 (3.3)
Guangdong	295 (14.3)
Guangxi	64 (3.1)
Hainan	5 (0.2)
Chongqing	30 (1.5)
Sichuan	86 (4.2)
Guizhou	19 (0.9)
Yunnan	18 (0.9)
Tibet	1 (0.0)
Shaanxi	51 (2.5)
Gansu	17 (0.8)
Qinghai	2 (0.1)
Ningxia	7 (0.3)
Xinjiang	13 (0.6)
