# Supplementary material for: Acceptance of COVID-19 Vaccination during the COVID-19 Pandemic in China
Source: Vaccines (Basel). 2020 Aug 27;8(3):482. doi: 10.3390/vaccines8030482 (PMC7565574; doi:10.3390/vaccines8030482)
Supplement: Supplementary file 1 [file vaccines-08-00482-s001.pdf]

vaccines

MDPI

Article

Acceptance of COVID-19 Vaccination during the COVID-19 Pandemic in China

Supplementary Materials

Table S1. Comparison of baseline characteristics between the vaccine demand group and vaccine delay group.

Items	Vaccine Demand Group N (%)	Vaccine Delay Group N (%)	*p*-Value
Age group			
18–25	186 (19.0)	253 (28.1)	
26–30	192 (19.6)	174 (19.4)	
31–40	272 (27.8)	216 (24.0)	
41–50	246 (25.1)	210 (23.4)	
51 and above	84 (8.6)	46 (5.1)	<0.001
Gender			
Female	501 (51.1)	521 (58.0)	
Male	479 (48.9)	378 (42.0)	0.003
Highest level of education			
Middle school and below	60 (6.1)	48 (5.3)	
High school	314 (32.0)	293 (32.6)	
Associate or bachelor	546 (55.7)	496 (55.2)	
Master and above	60 (6.1)	62 (6.9)	0.800
Marriage status			
Married	725 (74.0)	539 (60.0)	
Others (single, divorced or widowed）	255 (26.0)	360 (40.0)	<0.001
Location			
Central	265 (27.0)	224 (24.9)	
East	553 (56.4)	531 (59.1)	
West	162 (16.5)	144 (16.0)	0.483
Region			
Rural	210 (21.4)	178 (19.8)	
Urban	770 (78.6)	721 (80.2)	0.384
Employment status			
Employed	810 (82.7)	701 (78.0)	
Unemployed	170 (17.3)	198 (22.0)	0.011
Health status			
Good and above (good, very good)	754 (76.9)	653 (72.6)	
Fair or below (fair, poor, very poor)	226 (23.1)	246 (27.4)	0.032
Total family income in 2019			
≤ CNY 50,000 (USD 7246)	125 (12.8)	117 (13.0)	
CNY 50,000–100,000 (USD 7246–14,492)	254 (25.9)	236 (26.3)	
CNY 100,000–150,000 (USD 14,492–21,739)	233 (23.8)	238 (26.5)	
CNY 150,000–200,000 (USD 21,739–28,986)	180 (18.4)	154 (17.1)	
CNY 200,000–300,000 (USD 28,986–43,478)	115 (11.7)	102 (11.3)	
≥ CNY 300,000 (USD 43,478)	73 (7.4)	52 (5.8)	0.575
There are confirmed or suspected cases in the county			
Yes	723 (73.8)	702 (78.1)	
No or not clear	257 (26.2)	197 (21.9)	0.029
Perceived risk of infection			
High or very high	145 (14.8)	86 (9.6)	
Fair	265 (27.0)	263 (29.3)	
Low or very low	570 (58.2)	550 (61.2)	0.003
Pandemic impact on daily life			
Large or very large	679 (69.3)	588(65.4)	
Fair	233 (23.8)	214(23.8)	
Small or very small	68 (6.9)	97(10.8)	0.011
Pandemic impact on work			

Large or very large	663 (72.8)	580 (68.2)	
Fair	174 (19.1)	178 (20.9)	
Small or very small	74 (8.1)	92 (10.8)	0.066
Pandemic impact on income			
Large or very large	465 (55.6)	374 (52.2)	
Fair	233 (27.8)	194 (27.1)	
Small or very small	139 (16.6)	148 (20.7)	0.116
Received vaccination against influenza in the past season			
Yes	197 (20.1)	94 (10.5)	
No	783 (79.9)	805 (89.5)	<0.001
Refused vaccination of a certain type of vaccine in the past			
Yes	191 (19.5)	208 (23.1)	
No	789 (80.5)	691 (76.9)	0.053
COVID-19 vaccination is an effective way to prevent and control COVID-19			
Yes	919 (93.8)	812 (90.3)	
No	61 (6.2)	87 (9.7)	0.006
Doctor's recommendation is an important factor in vaccination decision-making			
Yes	880 (89.8)	704 (78.3)	
No	100 (10.2)	195 (21.7)	<0.001
Vaccine convenience (vaccination method, frequency, distance to vaccination sites, etc.) is an important factor in vaccination decision-making			
Yes	714 (72.9)	732 (81.4)	
No	266 (27.1)	167 (18.6)	<0.001
Vaccine price is an important factor in vaccination decision-making			
Yes	539 (55.0)	584 (65.0)	
No	441 (45.0)	315 (35.0)	<0.001

Notes: *p*-values of Chi-square test to check the significance of the differences among categories.

Table S2. Influencing factors on vaccination acceptance between the vaccine demand group and vaccine delay group, by including significant factors at the 10% level of the Chi-squared test into the multivariate logistic regression.

Characteristics	OR	SE	*p*-Value	95%CI
Age group				
18–25	Ref			
26–30	1.11	0.22	0.60	0.75–1.63
31–40	0.99	0.21	0.95	0.66–1.49
41–50	1.04	0.22	0.87	0.68–1.56
> 51	1.59	0.44	0.09	0.93–2.73
Gender				
Female	Ref			
Male	1.26	0.12	0.02	1.03–1.53
Marriage status				
Others (single, divorced or widowed）	Ref			
Married	1.67	0.25	<0.001	1.24–2.24
Employment status				
Unemployed	Ref			
Employed	0.98	0.20	0.90	0.66–1.45
Health status				
Fair or below (fair, poor, very poor)	Ref			
Good and above (good, very good)	1.13	0.13	0.28	0.90–1.42
There are confirmed or suspected cases in the county				
No or not clear	Ref			
Yes	0.70	0.08	<0.001	0.55–0.88
Perceived risk of infection				
Fair	Ref			
High or very high	1.46	0.25	0.03	1.05–2.04
Small or very small	1.01	0.11	0.94	0.81–1.26
Pandemic impact on daily life				
Fair	Ref			
Large or very large	1.00	0.12	0.97	0.79–1.28

Small or very small	0.66	0.14	0.04	0.44–0.99
Pandemic impact on work				
Fair	Ref			
Large or very large	1.05	0.14	0.70	0.81–1.37
Small or very small	0.82	0.17	0.35	0.55–1.24
Received vaccination against influenza in the past season				
No	Ref			
Yes	1.89	0.27	<0.001	1.43–2.50
Refused vaccination of a certain type of vaccine in the past				
No	Ref			
Yes	0.80	0.10	0.07	0.63–1.02
COVID-19 vaccination is an effective way to prevent and control COVID-19				
No	Ref			
Yes	1.55	0.29	0.02	1.08–2.23
Doctor's recommendation is an important factor in vaccination decision-making				
No	Ref			
Yes	2.25	0.3	<0.001	1.71–2.97
Vaccine convenience (vaccination method, frequency, distance to vaccination sites, etc.) is an important factor in vaccination decision-making				
No	Ref			
Yes	0.71	0.09	0.01	0.56–0.91
Vaccine price is an important factor in vaccination decision-making				
No	Ref			
Yes	0.75	0.08	0.01	0.61–0.92

2020 by the authors. Licensee MDPI, Basel, Switzerland. This article is an open access article distributed under the terms and conditions of the Creative Commons Attribution (CC BY) license (http://creativecommons.org/licenses/by/4.0/).
